# Case of Prolonged Gestation in Which the Date of Conception Was Accurately Ascertained

**Published:** 1848-08

**Authors:** 


*Case of Prolonged Gestation in which the date of Conception was accurately ascertained.* By R. H. McIlvain, M. D., Charlotte, N. C.

The following case of gestation, prolonged to probably 296, certainly to 293 days, occurred under the personal observation of the writer. The parties are of unexceptionable character, and the statement of the husband that no intercourse was had after the night of tne 4th of July, may be implicitly relied on.

Mrs. ————, whose character is above suspicion, was visited on the evening of July 1st, 1847, by her husband, whose business had compelled him to reside for more than a year before in a distant state. The husband remained till the morning of the 6th of July, and then departed, and did not return for more than nine months. On the nights of the 1st, 2nd, 3d, and 4th of July, there was sexual intercourse between the parties, but none on the night of the 5th or after. Shortly after Mrs. ——— considered herself pregnant, and on the 23d of April, 1848, was delivered, after an easy labor, of a fine healthy female child, weighing nine pounds.

Supposing impregnation to have occurred on the night of the first, as a consequence of the first coition, the duration of the pregnancy must have been 296 days, but if we suppose the last copulation to be the one from which the pregnancy resulted, the period of gestation was 293 days.

This case is interesting, inasmuch as it furnishes conclusive evidence, that gestation may be prolonged to thirteen, if not sixteen days beyond the usual period.

The large size of the child—being a full pound and a half above the average weight of female children, is a circumstance in favor of its having been carried beyond the usual period.

The mother had borne three children previously, none of which weighed over eight pounds.—*Am. Jour. Med. Sciences.*

CHARLOTTE, N. C., May 25, 1848.

